# Translations and Suggestions

**Published:** 1896-08

**Authors:** G. Leo Hagenburger

**Affiliations:** Brooklyn, N. Y.


On to Buffalo!

---

### TRANSLATIONS AND SUGGESTIONS.

By G. LEO HAGENBURGER, M.S., D.V.S.,
BROOKLYN, N. Y.

VETERINARIAN KNOLL has proved that out of twenty tuberculous cows killed at the abattoir at Prenzlau all of them were affected at the mammæ. *Walkering* has proved that the refractometer for the examination of butter is not to be relied upon in all cases, especially where the milk is taken from cows largely fed upon cotton-seed flour or oil-meal. Sell calls attention to the oleogrameter (in his report to the Kaiserlichen Gesundlsects Amt. of Prussia), the measurements showing various results of the same samples submitted for test under exactly the same conditions; clear butter shows less than oleomargarine; winter and summer butter also varied very largely. Brullé's method of using the nitrate of silver, test in order to ascertain if foreign oils have been added, is not what is claimed for it. Samples charged with vegetable oils have not reacted as indicated by the French scientist.

Veterinarian Pilarios, of Athens, Greece, shows in his statistics of three years 80 per cent. of cures effected with the repeated injection of mallein at intervals of eight to ten days. All animals so treated and recovered showed a rise of temperature after injection and had all the characteristic symptoms of glanders on clinical examinations beyond a doubt.

Why not isolate such patients in America, if valuable enough for treatment, especially when there is no indemnity now for the owner of such animals so affected?

Three deaths are reported in the district of Braunsberg, Halluponen, and Berlin, of grooms that were in attendance of glandered horses. All three succumbed to the malady.

A national law should be passed in America to prohibit the skinning of animals that are destroyed suffering from glanders, as the skin is not of sufficient value to endanger the lives of men working in rendering establishments where such cadavers are taken.

One hundred and nine deaths are reported in persons from anthrax or milzbrand; forty-three were butchers, four shepherds, six working at skinning of animals, the wife of one of the former, one groom, one hotelkeeper, two laborers on a farm, four handworkers in a horsehair weaving establishment.

Meat-poisoning caused severe attacks of stomach and intestinal catarrh and the death of a four-year-old child last spring in Canton Thurgan, Switzerland.

The meat ingested by many people of Thurgan was pickled and smoked pork, and was prepared from pigs which had suffered from a reddened condition of the skin and slight intestinal catarrh before they were killed. People after eating the above meat were taken with intense intestinal pain, diarrhœa, and muscular twitchings all over the body; all except the infant recovered; the latter was physically a strong and healthy child until it consumed some of the meat. This report shows clearly again the fallacy accepted by so many that curing, smoking, and boiling of pork will destroy and render inert pathogenic bacteria and lessen the virulence of toxin in the alimentary canal.

The Military Hospital Record shows the causation and transmission of typhoid fever (in the barracks of Schettstadt classes) by a milk hand who was employed in milking cows and bringing it to the station, whose son was suffering from the above disease. Strange that none of the civilian population in that city contracted the disease, which was prevented by cooking the milk properly before using it, which was not done by the men in the barracks.

A new test to detect a certain potassium and potassium bichromate compound which is used to adulterate and preserve milk gave very reliable results, and what is more desirable, under almost any climatic conditions and temperatures, namely, a yellowish-red color, if 1 c.cm. of the milk is mixed with 1 c.cm. of a 2 per cent. solution of nitrate of silver.

